# Generation of a set of genetically modified long QT syndrome induced pluripotent stem cell lines carrying knock-in variants rs120074178 (*KCNQ1* c.569G > A; p.Arg190Gln) and rs137854600 (SCN5A c.4865G > A; p. Arg1622Gln) and isogenic control lines

**DOI:** 10.1016/j.scr.2025.103755

**Published:** 2025-06-18

**Authors:** Nayara Sousa da Silva, Agnieszka D’Antonio-Chronowska, Reyna Hernandez-Benitez, Angelina Rose McCarron, Esra Karaca, Kai Fang, Juan Carlos Izpisua Belmonte, Athanasia D. Panopoulos, Keiichiro Suzuki, Kelly A. Frazer

**Affiliations:** a University of California, San Diego, Institute for Genomic Medicine, La Jolla, CA, USA; b University of California, San Diego, Department of Pediatrics, La Jolla, CA, USA; c Altos Labs, Inc., San Diego, CA, USA; d Board of Governors Regenerative Medicine Institute, Cedars-Sinai Medical Center, Los Angeles, CA, USA; e Department of Biomedical Sciences, Cedars-Sinai Medical Center, Los Angeles, CA, USA; f Institute for Advanced Co-Creation Studies, Osaka University, Toyonaka, Osaka, Japan; g Graduate School of Engineering Science, Osaka University, Toyonaka, Osaka, Japan; h Graduate School of Frontier Bioscience, Osaka University, Suita, Osaka, Japan

## Abstract

Long QT syndrome (LQTS) is an inherited channelopathy characterized by life-threatening arrhythmias. LQTS has many subtypes defined by the gene that contains the mutation, including LQT1 (*KCNQ1*), LQT2 (*KCNH2*), and LQT3 (*SCN5A*). Here, we used CRISPR/Cas9 technology to generate five isogenic human induced pluripotent stem cell (iPSC) lines, one line harboring an LQT1 variant rs120074178 (*KCNQ1* c.569G > A), two lines harboring an LQT3 variant rs137854600 (*SCN5A* c.4865G > A), and two derived control lines.

## 1. Resource utility

This set of isogenic iPSC lines edited for LQT1 or LQT3 causal variants and control lines can be differentiated into cardiomyocytes to model LQTS. These lines will provide an invaluable resource to study LQTS and in drug screens, especially for drug-induced LQT, and in gene therapy.

## 2. Resource details

Long QT syndrome (LQTS) affects 1 per 2000 people, with a 21 % mortality rate of untreated symptomatic patients. Known genetic variants underlie approximately 70 % of cases, with *KCNQ1* and *SCN5A* mutations accounting for 40–55 % and 5–10 % of genotype-positive cases, respectively (Schwartz, 2016). *KCNQ1* encodes the α-subunit of the Kv7.1 potassium channel, which is essential for a slow delayed rectifier current and proper cardiac repolarization. Missense *KCNQ1* variants are involved in the development of LQTS type 1 (LQT1) (Wu, 2016). *SCN5A* encodes the α-subunit of the Nav1.5 sodium ion channel, which is responsible for a synchronous and rhythmic repolarization. Variants in SCN5A that delay the repolarization phase are causative of LQT3 (Zaklyazminskaya,2016).

An iPSC line from the iPSCORE collection (Panopoulos, 2017) was employed as the parental line to study the genetic and physiological effects arising from missense variants in *KCNQ1* and *SCN5A* genes. The line was derived from dermal fibroblasts of a 25.7 year old Caucasian female (iPSCORE_3_2) using integration-free Sendai virus vectors (Table 1). Sendai virus clearance and loss of reprogramming genes was confirmed by RT-PCR (Panopoulos, 2017).

**Fig. 1.**
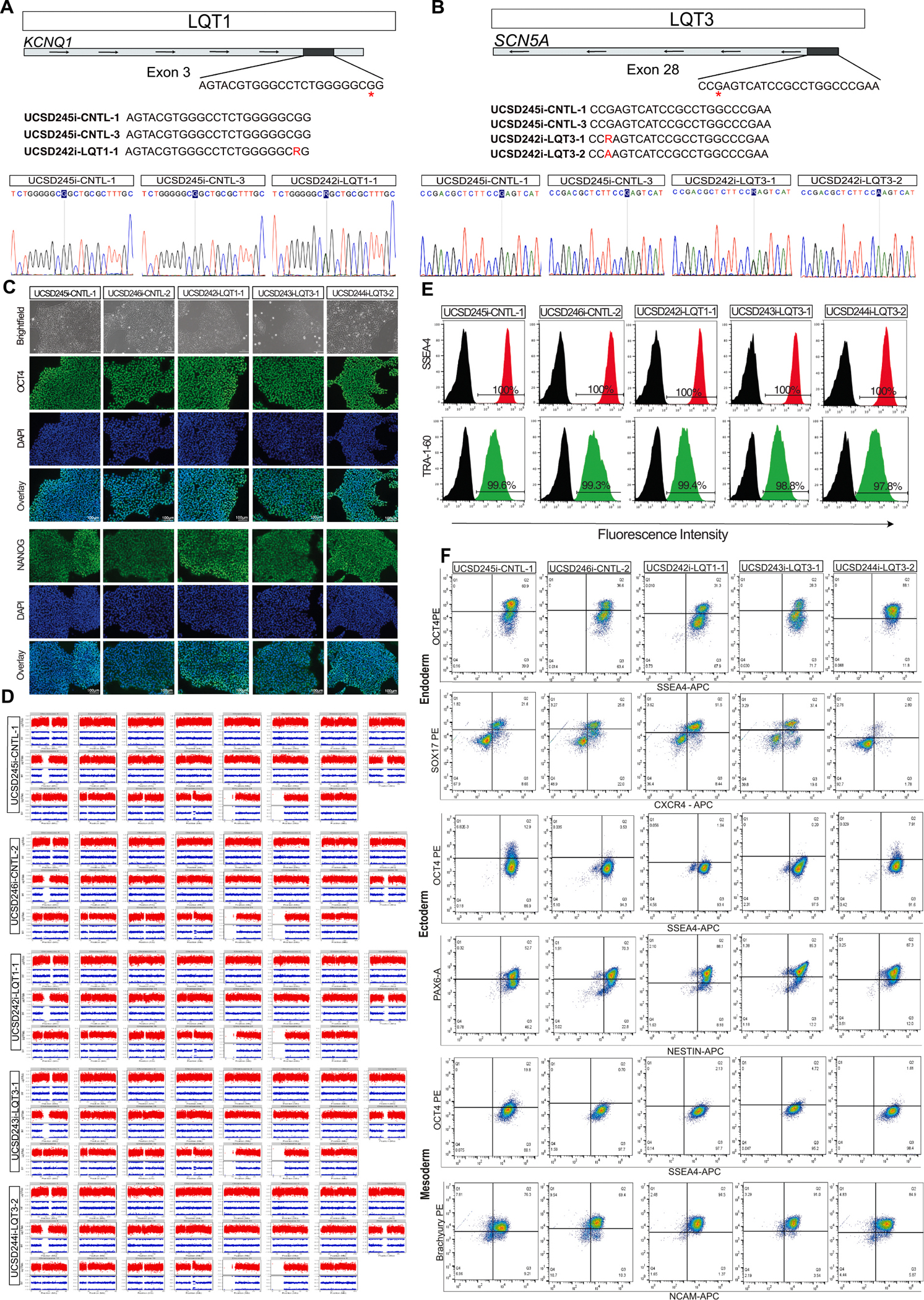


CRISPR/Cas9 technology was employed to generate three LQTS variant-carrying iPSC lines: one line (UCSD242i-LQT1–1) heterozygous for the *KCNQ1* missense variant rs120074178 (c.569G > A p. Arg190Gln); and two lines carrying the SCN5A missense variant rs137854600 (c.4865G > A; p.Arg1622Gln), one heterozygous (UCSD243i-LQT3–1) and one homozygous (UCSD244i-LQT3–2) (Fig. 1A and 1B). Additionally, isogenic controls were generated by exposing in parallel the parental line to the same transfection protocol (but without gene editing) and isolating two clones. After clonal selection, the presence of zygosity of the edited variants was verified by PCR on genomic DNA and Sanger Sequencing, and by the HumanCoreExome SNP array (Fig. 1A, 1B and Supplementary File 1).

The five iPSC lines displayed typical pluripotent stem cell morphology (Fig. 1C). Nuclear staining of pluripotency markers OCT4 and NANOG was confirmed by immunofluorescence in the iPSC lines (Fig. 1C). Normal karyotypes (46,XX) were confirmed by analyzing DNA isolated from the iPSC lines using the SNP array (Fig. 1D); which also identified a small 4.7 Mb chr20q duplication (chr20:29805100–34487478), inherited from the parental iPSCORE_3_2 iPSC line (Panopoulos, 2017). Sample identity of the iPSC lines was confirmed by comparing their SNP genotypes with the genotypes of blood DNA isolated from the donor (Supplementary File 2). Flow cytometry analysis demonstrated that more than 97 % of the cells expressed the pluripotent stem cell surface markers SSEA4 and TRA-1–60 (Fig. 1E).

The five isogenic iPSC lines successfully differentiated into the three germ layers, confirmed by the dual expression of CXCR4 and SOX17 (endoderm), NESTIN and PAX-6 (ectoderm), and Brachyury and NCAM (mesoderm) (Fig. 1F). A PCR-based assay confirmed that the lines were mycoplasma-free (Supplementary File 3). Furthermore, the fact that there were no random plasmid integration events was verified in the CRISPR-modified lines (Supplementary File 4). Additionally, off-target mutagenesis analysis of the top predicted sites showed no genomic alterations (Supplementary File 5). The cells have been deposited and are available for non-profit distribution through the WiCell biobank (https://www.wicell.org/home/stem-cells/catalog-of-stem-cell-lines/collections/nhlbi-next-gen-frazer.cmsx). For profit entities, please contact the corresponding author for access.

## 3. Materials and Methods

### 3.1. Cell culture

iPSC lines were cultured on Matrigel^®^(Corning)-coated plates using mTeSR^™^ 1 medium and passaged with Dispase II at 80 % confluency, as described in D’Antonio-Chronowska (2019).

### 3.2. Genome editing

iPSCs (1.5 × 10^7^) were dissociated with TrypLE (Invitrogen) and resuspended in 1 ml of MEF-conditioned medium containing 10 μM ROCK inhibitor Y-27632 (Biomol Inc.). Cells were electroporated with 15 μg of pCAG-1BPNLS-Cas9–1BPNLS-2AGFP, 15 μg of gRNAs (LQT1gRNA-mCherry or LQT3gRNA-mCherry, a modifed pCAGmCherry-gRNA) and 30 μg of single-strand oligonucleotides (LQT1 or LQT3; Table 2). After 48 h, cells were dissociated and GFP/mCherry double-positive cells were sorted and plated onto irradiated MEFs (Applied Stem Cell) pre-coated dishes. After 18 days, clones were transferred to 96-well plates and expanded. Isogenic controls were generated by exposing in parallel the parental iPSC line to the same transfection protocol but without gene editing. To generate the homozygous UCSD244i-LQT3–2 line, the procedure was repeated in the heterozygous UCSD243i-LQT3–1 line. iPSC line passage numbers are listed in Supplementary File 6.

### 3.3. Molecular karyotyping and genomic integrity

Genomic DNA was hybridized to Infinium HumanCoreExome-24 arrays (Illumina) and Copy Number Variation was called using Nexus CN (version 7.5), with B-allele frequencies and log R ratios manually inspected. Genetic identity was confirmed by comparing genotypes between the iPSC lines and donor blood-derived whole-genome sequence using the *genome* command in plink.

### 3.4. DNA extraction and sequencing

DNA was extracted and PCR amplification of CRISPR modification sites, off-target regions, and plasmid backbone sequences (to verify absence of integration) was performed using primers listed in Table 2.

### 3.5. Pluripotency status

For cell surface marker analysis of SSEA4 and TRA-1–60, cells were labeled with pluripotency markers (Table 2) in PBS containing 2 % FBS for 30 min at 4 °C, washed in 2 %FBS/PBS, and analyzed on the Sony ID7000 spectrum cell analyzer. Data were processed using FlowJo v10.10.0. For immunofluorescence analysis of OCT4 and NANOG, cells were first fixed in 4 % PFA for 15 min, permeabilized with 0.1 % Triton X-100 for 10 min and blocked with FBS 5 % for 1 h at room temperature (RT). Fixed cells were then stained overnight at 4 °C with primary antibody in 2 %FBS/PBS, before washing and staining for 1hr at RT in 2 %FBS/PBS containing secondary antibody (Table 2). Nuclei were stained with DAPI. Imaging was performed on an Echo Revolve Microscope.

### 3.6. In vitro trilineage differentiation assay

Cells were differentiated into three germ lineages using the STEMdiff Trilineage Differentiation Kit (StemCell Technologies). Cells were stained for flow cytometry and analyzed on the BD Accuri C6 Plus Flow Cytometer on the day of harvest. Cells were stained with surface antibodies (Table 2) in 10 % FBS/DMEM-F12 for 30 min at 4 °C, fixed in 2 % formaldehyde in PBS for 30 min at RT, and stained with internal antibodies (Table 2) in permeabilization Buffer for 60 min at RT. Data were analyzed using FlowJo v10.9.

### 3.7. Mycoplasma detection

Mycoplasma contamination was tested using the EZ PCR Mycoplasma Test Kit (Sartorius). All lines displayed only the 357 bp internal control band indicating they were negative.

## Supplementary Material

1

## Figures and Tables

**Table 1:** Characterization and validation.

Classification	Output type	Result	Data

**Schematic of a transgene/genetic modification**	Schematic illustrating the structure and location of the introduced genetic modification	N/A	Fig. 1A and 1B
**Morphology**	Photography	Bright-Field images show typical iPSC morphology	Fig. 1C
**Pluripotency status evidence for the described cell line**	Immunofluorescence	All lines expressed pluripotency markers OCT4 and NANOG	Fig. 1C
	Flow cytometry	TRA-1–60 and SSEA4 > 97 % positivity for all lines	Fig. 1E
**Karyotype**	Infinium HumanCoreExome SNP array	46 XX, Chr.20q duplication of approximately 4.7 Mb (chr20:29805100–34487478)	Fig. 1D
**Genotyping for the desired genomic alteration/allelic status of the gene of interest**	PCR across the edited site followed by	*KCNQ1* – c.569G > A; p.Arg190Gln	Fig. 1A and 1B
	Sanger Sequencing	*SCN5A* – c.4865G > A; p.Arg1622Gln	
	Evaluation of the – (homo-/hetero-/hemi-) zygous status of introduced genomic alteration(s)	Homo- or heterozygous alterations and isogenic controls confirmed	Fig. 1A, 1B and Supplementary file 1
	Transgene-specific PCR (when applicable)	N/A	N/A
**Verification of the absence of random plasmid integration events**	PCR of two regions of the plasmid backbone	No plasmid amplicon observed	Supplementary file 4
**Parental and modified cell line genetic identity evidence**	SNP array and WGS	The identities of the modified cell lines were certified by comparing SNP array genotypes with whole-genome sequence of blood DNA derived from the donor.	Supplementary file 2
**Mutagenesis / genetic modification outcome analysis**	PCR across the edited site follow by Sanger Sequencing	PCR targeting CRISPR edit sites.	Fig. 1A and 1B
	PCR-based analyses	N/A	N/A
	Southern Blot or WGS; western blotting (for knock-outs, KOs)	N/A	N/A
**Off-target nuclease activity analysis**	PCR across 5 of the top 10 predicted top likely off-target sites, whole genome/exome sequencing	No NHEJ-caused mutagenesis was observed for the predicted off target sites	Supplementary figure 5
**Specific pathogen-free status**	Mycoplasma	Mycoplasma testing by EZ PCR Mycoplasma Test Kit (Sartorius): Negative	Supplementary file 3
**Multilineage differentiation potential**	Trilineage differentiation assay (Flow cytometry)	All lines showed the ability to differentiate into all three germ layers.	Fig. 1F
**List of recommended germ layer markers**	Flow cytometry	Endoderm: Dual positivity of CXCR4, SOX17 ≥ 24.6 % Mesoderm: Dual positivity of BRACHYURY, NCAM ≥ 69.4 % Ectoderm: Dual positivity of NESTIN and PAX6 ≥ 42.5 %	Fig. 1F
***Outcomes of gene editing experiment (OPTIONAL)***	Brief description of the outcomes in terms of clones generated/establishment approach/ screening outcomes	N/A	N/A
***Donor screening (OPTIONAL)***	HIV 1 + 2 Hepatitis B, Hepatitis C	N/A	N/A
***Genotype – additional histocompatibility info (OPTIONAL)***	Blood group genotyping	N/A	N/A
	HLA tissue typing	N/A	N/A

**Table 2:** Reagents details.

Antibodies and stains used for immunocytochemistry/flow-cytometry			
	Antibody	Dilution	Company Cat # and RRID

Pluripotency Markers (Used for Pluripotency status)	Rabbit Anti-NANOG	1:100	Abcam Cat# ab21624RRID: AB_446437
Pluripotency Markers (Used for Pluripotency status)	Rabbit Anti-OCT4	1:100	Abcam Cat# ab19857RRID: AB_445175
Secondary Antibodies (Used for Pluripotency status)	Alexa Fluor 488 goat antirabbit IgG	1:1000	Invitrogen Cat# A11008RRID: AB_143165
Pluripotency Marker (Used for Pluripotency status)	PE anti-human SSEA4	1:20	Biolegend Cat# 330,405RRID: AB_1089207
Pluripotency Marker (Used for Pluripotency status	Alexa Fluor 488 anti-human TRA-1-60-R	1:20	Biolegend, Cat # 330,613RRID: AB_2295395
Pluripotency Markers (Used with Trilineage Differentiation Assay)	Mouse anti-OCT3/4	1:9	BD Bioscience Cat# 560,186RRID: AB_1645331
Pluripotency Markers (Used with Trilineage Differentiation Assay)	Mouse anti-Human SSEA4-APC	1:11	R&D systems Cat# FAB1435ARRID: AB_494994
Isotype control (Used for Trilineage Differentiation Assay)	IgG1-PE	1:9	BD Bioscience Cat# 559,320RRID: AB_397218
Endoderm Markers	Mouse anti-Human SOX17	1:17	BD Bioscience Cat# 561,591RRID: AB_10717121
Endoderm Markers	Mouse Anti-Human CXCR4	1:11	BD Bioscience Cat# 555,976RRID: AB_398616
Mesoderm Markers	Mouse Anti-Human Brachyury PE	1:17	R&D systems Cat# IC2085PRRID: AB_2271455
Mesoderm Markers	Human anti-NCAM-1/CD56 APC	1:21	R&D systems Cat# FAB2408ARRID: AB_562656
Ectoderm Markers	Human anti-PAX6 PE	1:9	R&D systems Cat# IC8150P
Ectoderm Markers	Mouse Anti-NESTIN	1:9	BD Bioscience Cat# 560,393RRID: AB_1645170
**Site-specific nuclease**			
Nuclease information	CAS9	pCAG-1BPNLS-Cas9-1BPNLS-2AGFP	
Delivery method	Electroporation		
Selection/enrichment strategy	FACS	GFP/mCherry double-positive cells	
Primers and Oligonucleotides used in this study		
	Target	Forward/Reverse primer (5–3)
Targeted mutation analysis/sequencing	CRISPR LQT1 edit (2,376 bp)	UCSD242i-LQT1-1, UCSD245i-CNTL-1 and UCSD245i-CNTL-3
		CTGTGTTTCCGTGGCCTTTTTC/ CAGTCCTTAGGGGACTCCATCT
	CRISPR LQT3 edit (858 bp)	UCSD243i-LQT3-1, UCSD244i-LQT3-2, UCSD245i-CNTL-1 and UCSD245i-CNTL-3
		GATCTGGAAGAGGCACAGCATG/ GAGCACTGATTTCTGGGCCAG
gRNA oligonucleotide	LQT1gRNA-mCherry	AGTACGTGGGCCTCTGGGGG, PAM: CGG
	LQT3gRNA-mCherry	TTCGGGCCAGGCGGATGACT, PAM: CGG
Off-target analysis/sequencing	**LQT1:**	CCATGCCATGCACATAAGGC / CAGCCAAGGGATTGCACCTA
	CNTN3 (874 bp)	TTCTGAGCCCAAAAGGCACT / TTGGGAAAAGTCTCCAACCCT
	FAM65B (506 bp)	CTCCTTCACCATGAGGAGCC/ AAAATCTACCCGCCGGAGTC
	Intergenic PPTC7-TCTN1	GTTAGGCGTGGTGTCTACCC / ATGGATCCACCCATGGCAAG
	(604 bp)	CTTTGACAACTTGCCCACAGC/ GGGAAAACCGAGTCTAGAACTTA
	Intergenic AL137019.1	GGTGGCCATCTTCCCCTACT/ GACCTTCCGCTGGACTCCTC
	(537 bp)	CGTGTAGATGGTCGAGGTGG/ CATGTCCCCGTAACCTACCG
	MFSD4 (508 bp)	TTGACACTCAGCAGCAAGGC/ GGTAAGTCCTGTTGGGGTCA
	**LQT3:**	TCCGAACCGCCATAAAAACTG/ CGAGTTGCTTTTGGCGACAT
	KCNA5 (583 bp)	CACCCCCTCAAGTTCCCAAG/ AACCCATTACACCTGGAGCC
	GALNT8/KCNA6 (687 bp)	
	MEGF9 (538 bp)	
	Intergenic RP11-17 M24.1 (554 bp)	
	Intergenic_RP11-435O5.7 (550 bp)	
Verification of the absence of random plasmid integration events	pCAG/m-Cherry (244 bp)	GCAACGTGCTGGTTATTGTG/ TTGGTCACCTTCAGCTTGG
	ORI/HUF-6 (669 bp)	GGGAAACGCCTGGTATCTTT/ GAGGGCCTATTTCCCATGATT
Genomic target sequence	KCNQ1	LQT1: chr11:2,570,719
	SCN5A	LQT3: Chr3:38,551,504
Bioinformatic gRNA on– and –off-target binding prediction tool used, specific sequence/outputs link(s)	CRISPOR (https://crispor.gi.ucsc.edu/)	LQT1: https://crispor.gi.ucsc.edu/crispor.py?batchId=MVrqXyXvvi0EiGkenOgP
		LQT3: https://crispor.gi.ucsc.edu/crispor.py?batchId=vSKYPIgdAJ8qhfLDKlXf
*ODNs/plasmids/RNA molecules used as templates for HDR-mediated site-directed mutagenesis*.	Gene-modified iPSCs	Oligonucleotide LQT1: ATGGAAATGGGCTTCCGGGCAAAGCGCAGCTGCCCCCAGAGGCCCACGTACTTGCTGCGG
		Oligonucleotide LQT3: AGAAGTACTTCTTCTCCCCGACGCTCTTCCAAGTCATCCGCCTGGCCCGAATAGGCCGCA

**Resource Table:** 

**Unique stem cell line identifier**	UCSDi001-A-1 https://hpscreg.eu/cell-line/UCSDi001-A-1 UCSDi001-A-2 https://hpscreg.eu/cell-line/UCSDi001-A-2 UCSDi001-A-3 https://hpscreg.eu/cell-line/UCSDi001-A-3 UCSDi001-A-4 https://hpscreg.eu/cell-line/UCSDi001-A-4 UCSDi001-A-5 https://hpscreg.eu/cell-line/UCSDi001-A-5
**Alternative name(s) of stem cell line**	UCSD242i-LQT1-1 UCSD243i-LQT3-1 UCSD244i-LQT3-2 UCSD245i-CNTL-1 UCSD246i-CNTL-2
**Institution**	University of California, San Diego
**Contact information of the reported cell line distributor**	Kelly A. Frazer – kafrazer@health.ucsd.edu
**Type of cell line**	iPSC
**Origin**	Human
**Additional origin info *(applicable for human ESC or iPSC)***	Age: 25.7 years Sex: Female Ethnicity: European The individual ID is iPSCORE_3_2
**Cell Source**	Dermal fibroblasts
**Method of reprogramming**	Non-integrating, Sendai virus
**Clonality**	Clonal/ Sanger sequencing (Panopoulos, 2017)
**Evidence of the reprogramming transgene loss (including genomic copy if applicable)**	RT-PCR (Panopoulos, 2017)
**The cell culture system used**	MEF-conditioned medium/ Irradiated mouse embryonic fibroblasts during the CRISPR stage mTeSR 1 medium/Matrigel-coated plates, after knock-in mutation
**Type of the Genetic Modification**	Induced modification at *SCN5A* or *KCNQ1* genes by CRISPR mediated editing
**Associated disease**	UCSD242i-LQT1-1: 192,500 – Long QT syndrome 1, LQT1 UCSD243i-LQT3-1 and UCSD244i-LQT3-2: 603,830 – Long QT syndrome 3, LQT3
**Gene/locus modified in the reported transgenic line**	UCSD242i-LQT1-1: rs120074178; Hg38 chr11:2,570,719 UCSD243i-LQT3-1 and UCSD244i-LQT3-2: rs137854600; Hg38 chr3:38,551,504
**Method of modification / user-customisable nucleases (UCN) used, the resource used for design optimisation**	CRISPR/Cas9
**User-customisable nuclease (UCN) delivery method**	N/A
**All double-stranded DNA genetic material molecules introduced into the cells**	pCAGmCherry
**Evidence of the absence of random integration of any plasmids or DS DNA introduced into the cells.**	PCR targeting plasmid backbone
**Analysis of the nuclease-targeted allele status**	PCR and Sanger Sequencing
**Homozygous allele status validation**	HumanCoreExome SNP array, PCR and
**Method of the off-target nuclease activity prediction and surveillance**	Sanger sequencing Region-specific PCR and Sanger Sequencing of the top predicted off-target sites
**Descriptive name of the transgene**	N/A
**Eukaryotic selective agent resistance cassettes (including inducible, gene/cell type-specific)**	N/A
**Inducible/constitutive expression system details**	N/A
**Date archived/stock creation date**	April 2023
**Cell line repository/bank**	https://www.wicell.org/home/stem-cells/catalog-of-stem-cell-lines/collections/nhlbi-next-gen-frazer.cmsx
**Ethical/GMO work approvals**	This study was approved by IRB committee at the University of California San Diego and The Salk Institute, IRB # 110776ZF
**Addgene/public access repository recombinant DNA sources’ disclaimers (if applicable)**	pCAGmCherry-gRNA (Addgene 87110) pCAG-1BPNLS-Cas9-1BPNLS-2AGFP (Addgene 87109)

## Data Availability

Data will be made available on request.

## Appendix A. Supplementary data

Supplementary data to this article can be found online at https://doi.org/10.1016/j.scr.2025.103755.
