# Supplementary material for: Generation of a set of genetically modified long QT syndrome induced pluripotent stem cell lines carrying knock-in variants rs120074178 (KCNQ1 c.569G > A; p.Arg190Gln) and rs137854600 (SCN5A c.4865G > A; p. Arg1622Gln) and isogenic control lines
Source: Stem Cell Res. Author manuscript; Available in PMC 2026 May 30. (PMC13222054; doi:10.1016/j.scr.2025.103755)
Supplement: 1 [file NIHMS2168660-supplement-1.pdf]

Supplementary file 1: To confirm that UCSD244i-LQT3-2 is homozygous and not hemizygous as a result of allelic dropout, we examined the HumanCoreExome SNP array data spanning approximately 500kb upstream and downstream of the CRISPR modification site. A. The four closest heterozygous SNPs to the target site were selected, and their locations and genotypes are shown below. UCSD244i-LQT3-2 retains the same heterozygous SNPs as the isogenic control lines and the heterozygous CRISPR-modified line UCSD243i-LQT3-1, which would not be observed if the line was hemizygous in this interval. B. Log R Ratio and B Allele Frequency (BAF) plots indicate no alterations in copy number or allele frequency in UCSD244i-LQT3-2, supporting that the homozygous genotype was achieved through successful CRISPR editing. However, due to the distance of the nearest heterozygous SNPs from the edited region and the resolution limits of the SNP array for detecting copy number alterations, the possibility of a small deletion cannot be entirely ruled out.

A

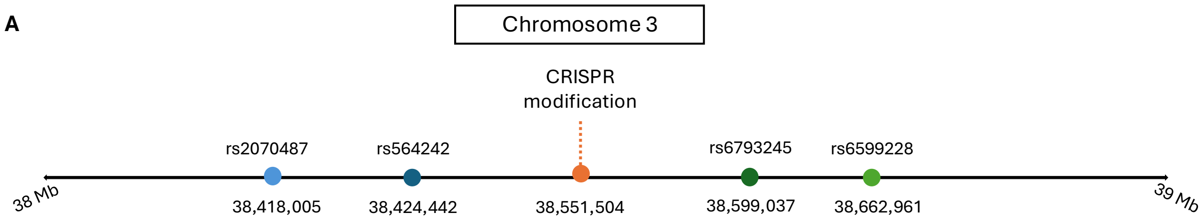

	SNP array A/B alleles			
CELL ID	rs2070487	rs564242	rs6793245	rs6599228
UCSD243i-LQT3-1	[T/C]	[A/G]	[A/G]	[T/C]
UCSD244i-LQT3-2	[T/C]	[A/G]	[A/G]	[T/C]
UCSD245i-CNTL-1	[T/C]	[A/G]	[A/G]	[T/C]
UCSD246i-CNTL-2	[T/C]	[A/G]	[A/G]	[T/C]

B

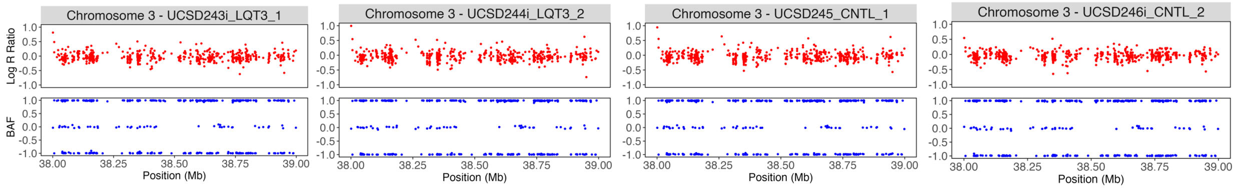

Supplementary file 2: Genetic identity was confirmed by matching genotypes obtained from SNP arrays hybridized with DNA derived from the iPSC lines and whole genome sequences of DNA derived from the donor's blood using the *genome* command in plink. All iPSC lines had a PI HAT > 0.99 indicating a match.

Cell ID	PI HAT
UCSD242i-LQT1-1	1
UCSD243i-LQT3-1	1
UCSD244i-LQT3-2	1
UCSD245i-CNTL-1	1
UCSD246i-CNTL-2	0.9999

Supplementary file 3: Mycoplasma test results are shown for each iPSC line. Yellow boxes indicate the result for the line specified at the top of the image. Positive control (PC) samples exhibit both a 270bp and a 357bp band, while negative control (NC) samples displayed only the 357bp internal control band. All IPCS lines tested negative for Mycoplasma.

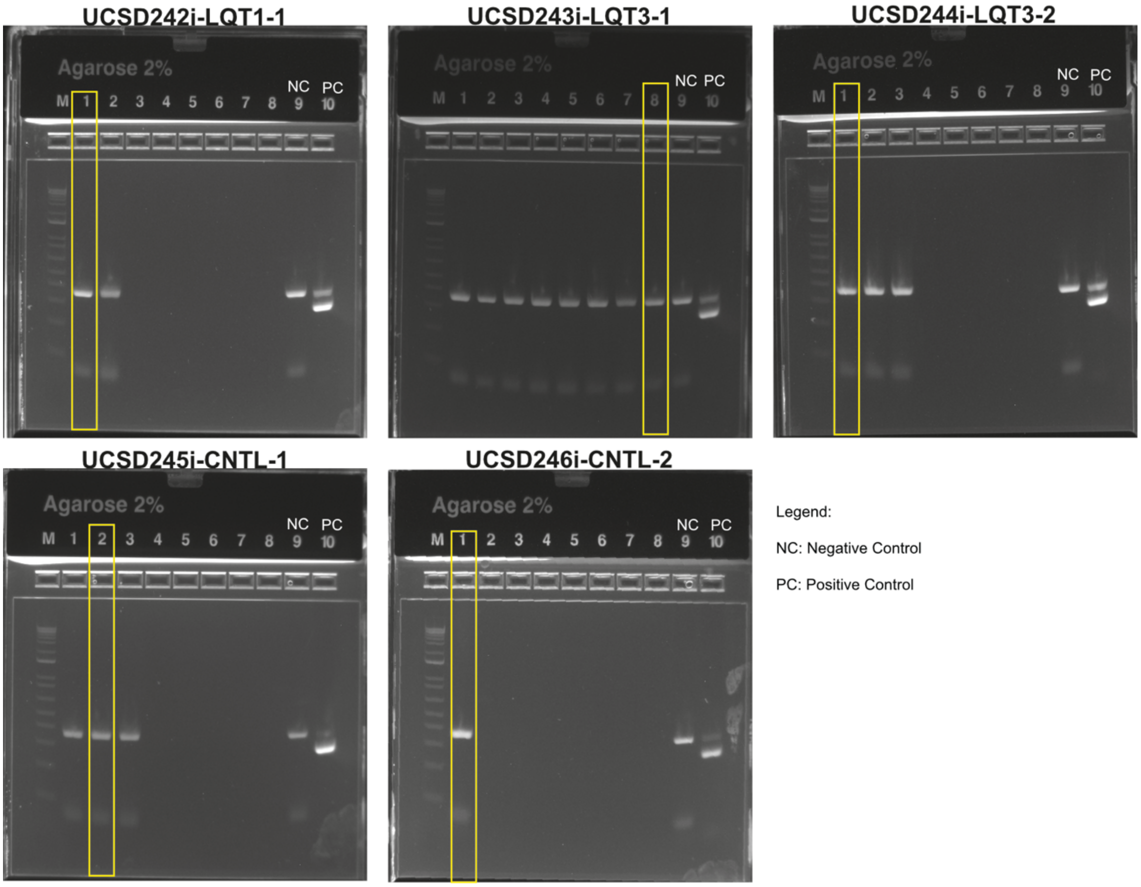

Supplementary file 4: To assess the absence of random plasmid integration events in the five iPSC lines, we performed PCR targeting of two CAG-mCherry plasmid backbone regions using the primer sets listed in Table 2: pCAG-F/mCherry-R (244 bp) and HUF-6-F/ORI-R (669 bp). No plasmid-specific bands were detected in any of the five iPSC lines, indicating a lack of integration events.

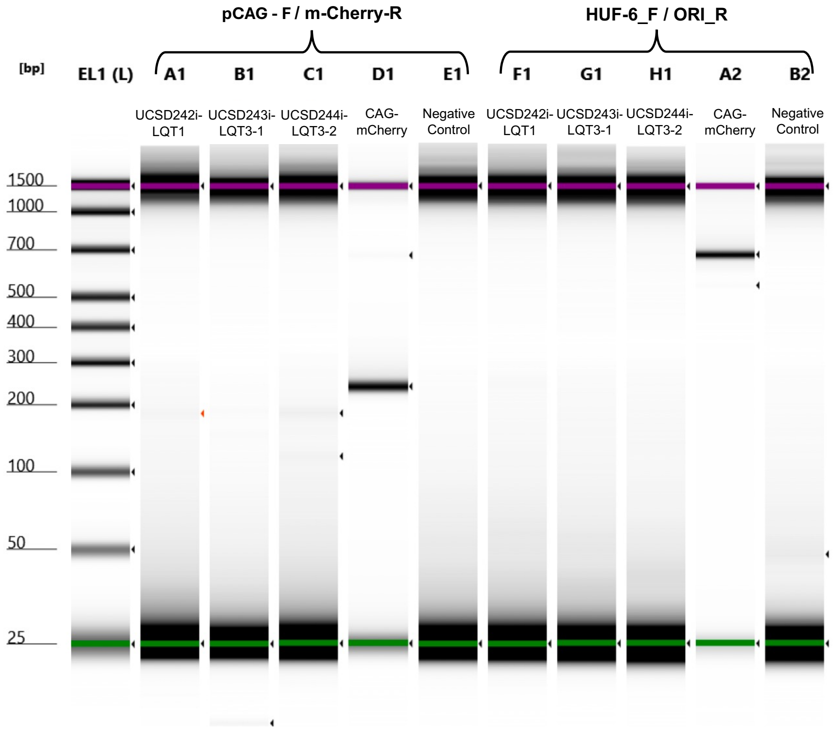

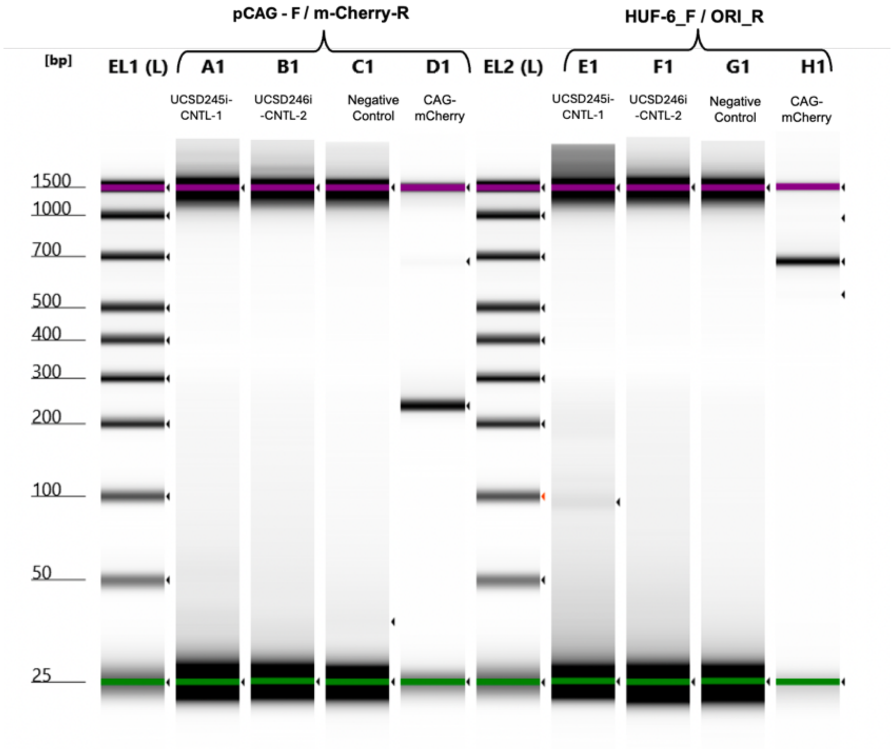

Supplementary file 5: The CRISPOR program was used to identify the top 10 predicted off-target sites for each of the LQT edits: LQT1 (A) and LQT3 (B). Five of the top 10 predicted off target sites were PCR amplified (primers in Table 2) and Sanger Sequenced by Azenta Life Sciences. The trace files showed identical sequences between edited and isogenic control lines, confirming no mutagenesis at predicted off-target sites.

A.

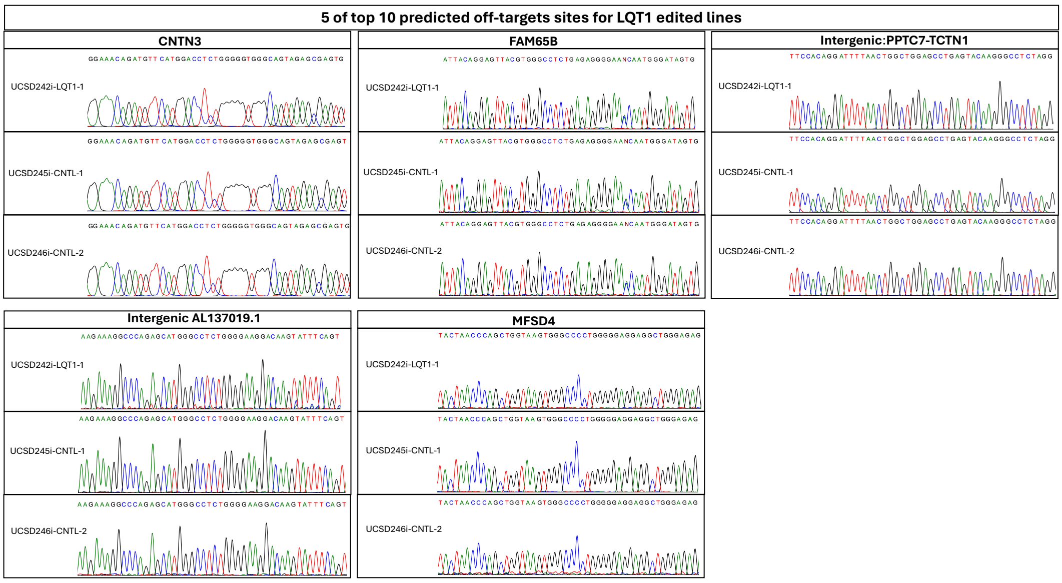

B.

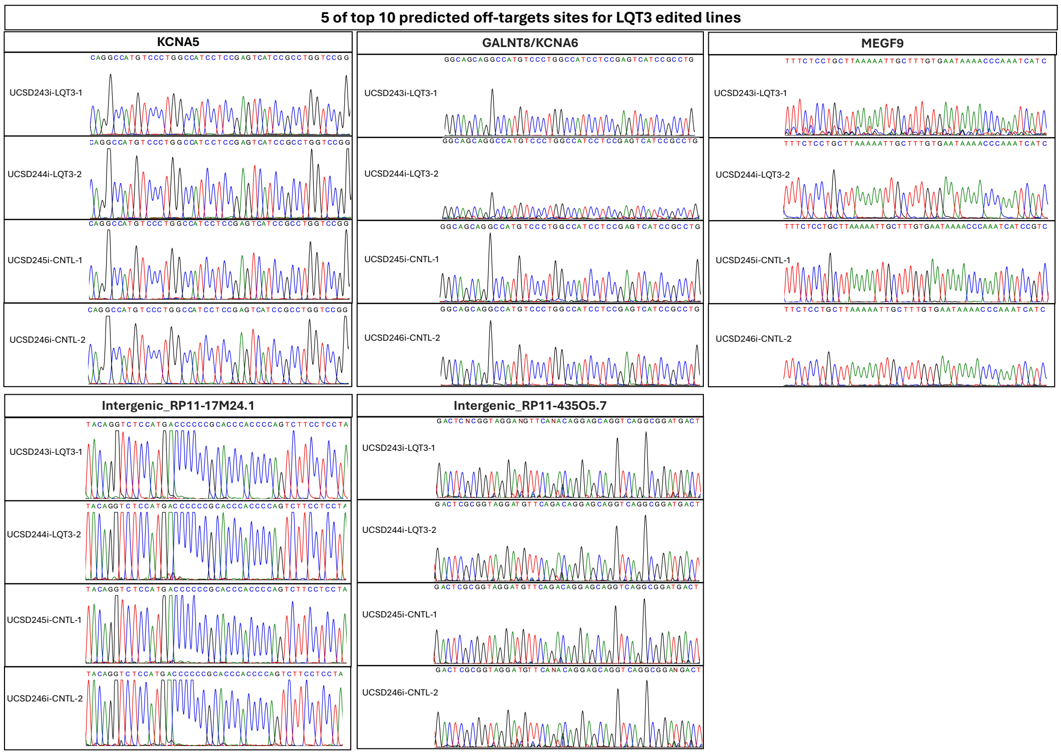

Supplementary file 6: Passage numbers of iPSC lines used in all assays described in this paper.

Cell ID	Passage number
UCSD242i-LQT1-1	P17
UCSD243i-LQT3-1	P15
UCSD244i-LQT3-2	P13
UCSD245i-CNTL-1	P15
UCSD246i-CNTL-2	P16
